# Psychosocial factors and overall mortality after prostate cancer diagnosis among predominantly low-income Black and White adults

**DOI:** 10.1093/jncics/pkag029

**Published:** 2026-05-05

**Authors:** Douglas DeMoulin, Loren Lipworth, Melinda C Aldrich, Leo J Russo, Heather Munro, Francesca Kolitsopoulos, Ronald Fisa, Kabisa Mwala, Martha J Shrubsole, Wei Zheng, Xiao-Ou Shu

**Affiliations:** Division of Epidemiology, Department of Medicine, Vanderbilt University Medical Center, Nashville, TN, United States; Division of Epidemiology, Department of Medicine, Vanderbilt University Medical Center, Nashville, TN, United States; Division of Epidemiology, Department of Medicine, Vanderbilt University Medical Center, Nashville, TN, United States; Global Medical Epidemiology, Research & Development, Pfizer, Inc, Collegeville, PA, United States; Division of Epidemiology, Department of Medicine, Vanderbilt University Medical Center, Nashville, TN, United States; Clinical Data Sciences, Real World Evidence, Gilead Sciences, Inc., Parsippany, NJ, United States; School of Medicine and Public Health, University of Zambia, Lusaka, Zambia, Africa; School of Medicine and Public Health, University of Zambia, Lusaka, Zambia, Africa; Division of Epidemiology, Department of Medicine, Vanderbilt University Medical Center, Nashville, TN, United States; Division of Epidemiology, Department of Medicine, Vanderbilt University Medical Center, Nashville, TN, United States; Division of Epidemiology, Department of Medicine, Vanderbilt University Medical Center, Nashville, TN, United States

## Abstract

**Background:**

Psychosocial factors may influence prostate cancer (PCa) survival, but evidence is limited for low-income populations.

**Methods:**

This study used data from the Southern Community Cohort Study (SCCS), a prospective cohort of ∼85 000 individuals aged 40-79, from predominantly low-income backgrounds, enrolled between 2002 and 2009 across 12 southeastern states. A total of 1367 men (1058 Black and 309 White) developed PCa during the follow-up of 34 313 men were included. The Kaplan-Meier method was used to generate 5-year survival rates with log-rank tests for statistical significance. Multivariable Cox models were used to estimate adjusted hazard ratios (aHRs) and 95% confidence intervals (CIs) for psychosocial-mortality associations.

**Results:**

Over 18 years of follow-up, 291 Black (27.5%) and 59 White (19.1%) PCa patients died (*P* = .03). White men self-reporting major depressive symptoms and their inability to control important things in life (ITC) had an 11% (*P*_survival_ = .01) and 12% (*P*_survival_ = .01) lower 5-year survival compared with those who did not, with aHR of 2.56, (95% CI = 1.13 to 5.77) and 2.30 (95% CI = 1.07 to 4.95), respectively. Inverse associations were found for per SD increase in depression score (aHR = 0.84, 95% CI = 0.74 to 0.96) among Black patients. Testing for multiplicative interaction was significant for race-depression (*P*_interaction_ = .02) and race-ITC (*P*_interaction_ = .04). The associations were mainly seen among PCa cases diagnosed within 5 years after baseline survey.

**Conclusion:**

Psychosocial-mortality associations among PCa patients are complex and may not affect individuals equally. Programs aiming to reduce mortality for individuals with PCa should consider their psychosocial needs and demographic background.

## Introduction

Prostate cancer (PCa) incidence and mortality represents a national and global burden,^1^^,^^2^ with approximately 313 780 new cases and 35 770 deaths reported in the United States.^1^ Racial disparities in PCa persist over time in the United States.^3^ In comparison to White men, Black men have a higher incidence of PCa across all cancer stages,^4^ have lower survival rates,^4^ have fewer screenings using prostate-specific antigen (PSA),^5^^,^^6^ are diagnosed with PCa at an earlier age, and received less initial treatment.^7^ Although mental health disparities are poorly understood, mental health symptoms are worse among men with PCa compared with men without PCa.^8^

Diagnosing mental health conditions, such as depression, has important implications for PCa survival.^8^^,^^9^ Men with PCa and comorbid depressive symptoms are likely to exhibit “lower adherence to treatment, more prolonged hospitalizations, more adverse reactions to treatment, and lower quality of life.”^8^^,^^10^ Conversely, mental health factors may augment one’s quality of life and possibly improve survival. For example, studies have reported spiritual well-being, religious coping, and quality of life as factors that positively influence overall survival.^11^^,^^12^ However, studies also report inverse relationships with fatigue during cancer treatment, mental health (namely, depression),^13^ and not being married, adversely influence cancer mortality.^14^ Mobility limitations affected church attendance more in Black men (30.1%) than White men (14.7%).^15^ In 2018, Dickey et al.^16^ developed a schematic model based on their findings from an integrated review that described positive and negative impacts on PCa survival in 4 domains: religion and spiritual well-being, social well-being, physical/functional well-being, and psychosocial well-being.

Despite the existence of racial disparities in the burden and survival of PCa, limited studies have explored the role of psychosocial factors in these survival disparities in a predominately low-income population. The aim of this prospective study was to evaluate psychological factors, including depression, social support, and spiritual and religious beliefs, on overall survival among Black and White men from a low-income population, diagnosed with PCa.

## Methods

### Study population and design

This study analyzed data collected on men who developed incident PCa, identified via linkages with 12 state cancer registries up to December 31, 2019, from the Southern Community Cohort Study (SCCS). The SCCS is a prospective cohort study that enrolled ∼85 000 men and women between 2002 and 2009 across 12 Southern US states—Alabama, Arkansas, Florida, Georgia, Kentucky, Louisiana, Mississippi, North Carolina, South Carolina, Tennessee, Virginia, and West Virginia—and followed them for cancer incidence and mortality. Recruitment occurred primarily in Community Health Centers (CHCs ∼85%), with a small percentage by mail (for the general population, ∼15%).^17^^,^^18^ Participants recruited from CHCs completed in-person interviews and participants enrolled from the general population completed a mailed questionnaire containing the same questions to capture information on sociodemographic, behavioral, and lifestyle factors; medical history; and psychosocial variables. By design, a majority of the participants were low-income adults, providing an exceptional opportunity to investigate the causes of cancer and other health disparities among understudied populations.

Cancer incidence and mortality were ascertained through linkages with state cancer registries and the National Death Index. A more detailed description of this cohort can be found elsewhere.^17^^,^^18^ All participants provided written, informed consent and the SCCS was approved by the Institutional Review Boards of Vanderbilt University Medical Center and Meharry Medical College.

### Exposure assessment of psychosocial factors

*Depressive Symptoms* were assessed using the Center for Epidemiologic Studies Depressive Scale (CESD)–10 which captures self-reported depressive symptoms over the past week.^19^ The CESD-10 measures depressed mood, feelings of guilt, worthlessness, helplessness, psychomotor retardation, loss of appetite, and sleep difficulties.^18^ Item responses range from 0 to 30 and are rated on a 4-point Likert scale as follows: “Rarely or none of the time” (< 1 day), “Some or a little of the time” (1-2 days), “Occasionally or a moderate amount of time” (3-4 days), and “All of the time” (5-7 days), with higher scores indicating greater severity of depressive symptoms.^19^ Depressive symptoms were categorized as follows: no depressive symptoms (0-9), probable depressive symptoms^19^ (10-15), and major depressive symptoms (≥16).

*Social Support* was ascertained using 2 questions: “How many close friends or relatives would help you with your emotional problems or feelings if you needed it?” and “How many people could you ask for help in an emergency or with lending you money?” Support structures were categorized as <2 people and ≥2 people.

*Religiosity/Spirituality* were ascertained through the following 3 questions: (1) Comfort in faith: “How much is religion, faith, or God a source of strength and comfort?” which was categorized as “a great deal,” “quite a bit,” and “not very much/somewhat.” (2) Spirituality: “How spiritual or religious do you consider yourself to be?” which was categorized as “very,” “fairly,” and “slightly/not at all.” (3) Attending faith-based service: “How often do you attend religious or faith services during the year?” which was categorized as “< once per week,” and “≥ once per week.”

*Participants’ ability to deal with stress* was assessed by 2 questions: “In the past month, how often have you felt that you were unable to control the important things in your life?” which was categorized as “rarely/none of the time,” “some of the time,” and “much/most/all of the time” and “In the past month, how often have you felt difficulties were piling up so high that you could not overcome them?” which was categorized as “rarely/none of the time,” “some of the time,” and “much/most/all of the time.”

### Statistical analysis

Baseline characteristics were described using descriptive statistics. Shapiro-Wilk’s test of normality assessed all continuous variables (ie, age at cancer diagnosis, time interval between cancer diagnosis and death, and pack-years smoking) to confirm underlying distributions. Mann-Whitney *U* tests were used for non-normally distributed data to test median differences of variables under study. Chi-square and Fisher’s exact tests examined differences in binary and categorical variables for psychosocial factors. Five-year survival was estimated using the Kaplan-Meier method, while statistical significance was assessed using the log-rank test. Multivariable Cox proportional hazards models, with time-to-event as the timescale, were applied to estimate adjusted hazard ratios (aHRs) and 95% confidence intervals (CIs) for the associations between psychosocial factors and risk of total mortality. All models were adjusted for age at PCa diagnosis, time interval from baseline enrollment to cancer diagnosis, education (<high school, high school, >high school), income (<$15 000, ≥$15 000), cancer stage (localized, regional/distant, unknown/unstaged), PCa treatment (no, yes), comorbidity using the Charlson Comorbidity Index (0, 1, 2+ comorbidities), ever having had a PSA test (no, yes), and enrollment source (clinic, mailed) for initially adjusted models, and then further adjusted for physical activity (no, yes), ever smoking (no, yes), and daily alcohol consumption. These covariates were selected based on previous publication of their associations with depression and mortality. Tests for trend treated categorical variables as continuous for depressive symptoms. Depressive symptoms were assessed continuously in per SD increase among Black (SD = 4.94) and White individuals (SD = 5.68), with a test for interactions by race. Race specific depression-mortality associations were further adjusted for other psychosocial factors to evaluate whether the latter could mitigate the association. Additional analyses were stratified by time window between study enrollment and PCa diagnosis (<5 years and ≥5 years). All statistical tests were based on 2-tailed probability and a significance level set at α < 0.05. All analyses were performed using SAS Enterprise statistical software version 7.1.

## Results

This study included 1058 Black and 309 White men with PCa from the SCCS. Total mortality after PCa diagnosis was higher for Black men (27.5%) than for White men (19.1%) (*P* = .003). Men with PCa were more likely to die with lower income, not married or living with a partner, with a prior history of smoking, and having smoked more pack-years of cigarettes. Within each racial group, Black men were more likely to die if they had less than a high school education, had a regional/distant cancer stage, or received no treatment for PCa, whereas White men were more likely to die if they had no PSA tests, no physical activity, or no health insurance. All these differences were statistically significant (Table 1).

**Table 1.** Demographics and characteristics of the study population by survival status and race (*n* = 1367).

	Black			White		
	Alive	Died	*P*	Alive	Died	*P*
Race (No., %)	767 (72.50)	291 (27.50)	–	250 (80.91)	59 (19.09)	–
Age of diagnosis (years, M ± SD)	61.82 ± 7.16	63.84 ± 7.74	**<.001**	65.93 ± 6.83	66.49 ± 6.97	.966
Time interval of enrollment to cancer diagnosis (months, M ± SD)	92.01 ± 52.04	64.64 ± 46.17	**<.001**	82.64 ± 48.16	52.15 ± 35.56	**<.001**
Education						
<High school	217 (28.29)	134 (46.05)	**<.001**	24 (9.60)	11 (18.64)	.110
High school	300 (39.11)	89 (30.58)		72 (28.80)	18 (30.51)	
>High school	250 (32.59)	68 (23.37)		154 (61.60)	30 (50.85)	
Income						
<$15 000	351 (45.76)	182 (62.54)	**<.001**	55 (22.00)	24 (40.68)	**.003**
≥$15 000	416 (54.24)	109 (37.46)		195 (78.00)	35	
Marital status						
Married/partnered	361 (47.07)	108 (37.11)	**.004**	201 (80.40)	32 (54.24)	**<.001**
Separated/divorced/widowed/single	406 (52.93)	183 (62.89)		49 (19.60)	27 (45.76)	
PSA ever						
No	323 (42.11)	117 (40.21)	.574	56 (22.40)	24 (40.68)	**.004**
Yes	444 (57.89)	174 (59.79)		194 (77.60)	35 (59.32)	
Enrollment source						
Clinic	647 (84.35)	269 (92.44)	**<.001**	88 (35.20)	34 (57.63)	**.002**
Mailed	120 (15.65)	22 (7.56)		162 (64.80)	25 (42.37)	
Ever smoke						
No	242 (31.55)	52 (17.87)	**<.001**	94 (37.60)	11 (18.64)	**.006**
Yes	525 (68.45)	239 (82.13)		156 (62.40)	48 (81.36)	
Pack-years smoked [*N* (mean ± SD)]	495 (20.33 ± 20.30)	232 (23.82 ± 20.57)	**.004**	141 (28.44 ± 23.93)	45 (46.33 ± 39.54)	**.001**
Daily alcohol consumption						
No	298	102	.255	100	22	.702
Yes	469 (3.27 ± 5.49)	189 (4.31 ± 7.28)	.220	150 (1.40 ± 2.40)	37 (2.69 ± 4.46)	.133
Physical activity						
No	534 (69.62)	220 (75.60)	.055	154 (61.60)	48 (81.36)	**.004**
Yes	233 (30.38)	71 (24.40)		96 (38.40)	11 (18.64)	
No. of comorbidities						
0 to 1	385 (50.20)	143 (49.14)	.759	141 (56.40)	26 (44.07)	.087
2+	382 (49.80)	148 (50.86)		109 (43.60)	33 (55.93)	
Stage						
Localized	608 (79.3)	200 (68.7)	**<.001**	202 (80.80)	47 (79.66)	.490^⁋^
Regional/distant	88 (11.5)	64 (22.0)		36 (14.4)	7 (11.9)	
Unstaged	71 (9.2)	27 (9.3)		12 (4.8)	5 (8.5)	
Prostate cancer treatment						
No	205 (26.73)	103 (35.40)	**.020**	58 (23.20)	20 (33.90)	.209
Yes	519 (67.67)	172 (59.11)		150 (60.00)	29 (49.15)	
Unknown	43 (5.61)	16 (5.50)		42 (16.80)	10 (16.95)	
Insurance						
No	282 (36.77)	108 (37.11)	.917	31 (12.40)	14 (23.73)	**.027**
Yes	485 (63.23)	183 (62.89)		219 (87.60)	45 (76.27)	

Chi-square and Fisher’s exact (^¶^) tests were used to compare categorical variables, and Mann-Whitney *U* tests were used to compare continuous variables; *N* reported pack-years among ever-smokers, and *P* values reported comparing daily alcohol consumption assessed binary and by daily consumption (yes). Abbreviation: PSA = prostate specific antigen. Bold indicates the association reached statistical significance.

The 5-year overall survival rate among Black and White men with PCa by psychosocial factors are presented in Table 2. Most psychosocial factors were not significantly associated with overall mortality. The exception was White patients with no self-reported depression whose 5-year survival rate was 91%, compared with 79% and 80% for those with self-reported probable or major depression scores, respectively (*P*_survival_ = .007). Additionally, White patients who reported “much, most, or all the time” for their inability to control important things in life (ITC) had a survival rate of 80%, compared with 92% among those self-reporting their ITC as “rarely or none of the time” (*P*_survival_ = .014).

**Table 2.** Psychosocial factors and 5-year survival rate among men with prostate cancer.

	Black				White			
	Total	Events	5-year survival rate	*P* (Survival)	Total	Events	5-year survival rate	*P* (Survival)
Depression								
No depressive symptoms	722	209	77%	.337	220	34	91%	**.007**
Probable depressive symptoms	236	60	84%		42	11	79%	
Major depressive symptoms	72	17	81%		26	11	80%	
Help during emergencies								
≥2 people	784	213	80%	.226	240	46	88%	.956
<2 people	260	74	76%		57	12	91%	
Support (friends/relatives)								
≥2 people	835	234	79%	.788	242	43	88%	.228
<2 people	210	54	80%		57	14	91%	
Inability to control important things in life (ITC)								
Rarely or none of the time	573	174	77%	.360	167	23	92%	**.014**
Some of the time	345	84	81%		97	19	87%	
Much, most, or all of the time	129	32	82%		40	14	80%	
Difficulties of things piling on and not overcoming them								
Rarely or none of the time	583	175	78%	.604	187	29	91%	.079
Some of the time	312	78	81%		67	13	83%	
Much, most, or all of the time	129	33	81%		36	12	87%	
Comfort in faith								
A great deal	702	196	80%	.954	136	24	89%	.767
Quite a bit	247	67	79%		67	15	86%	
Not very much/somewhat	102	19	76%		102	19	91%	
Attending faith-based services								
<Once per week	494	147	77%	.052	175	37	88%	.645
≥Once per week	554	143	81%		132	22	90%	
Spirituality								
Very	537	145	78%	.250	117	21	90%	.486
Fairly	381	99	81%		132	24	89%	
Slightly/not at all	130	45	78%		58	14	86%	

Kaplan-Meier curves used to determine survival rates; *P* values for the survival rates were measured using log-rank test. Abbreviation: ITC = inability to control important things in life. Bold indicates the association reached statistical significance.

The associations between psychosocial factors and total mortality by race with adjustment for potential confounders are presented in Table 3. In the initial model, Black men who self-reported attending faith-based services at least once weekly had reduced total mortality compared with Black men who did not (aHR = 0.77, 95% CI = 0.61 to 0.98). The association was no longer significant after controlling for physical activity, smoking, and daily alcohol consumption (aHR = 0.84, 95% CI = 0.66 to 1.08). Among Black men, there was an inverse association per standard deviation (4.94) increase of depressive scores in fully adjusted models (aHR = 0.84, 95% CI= 0.74 to 0.96), but no significant associations were noted when depression was analyzed categorically, although a significant trend was noted (*P*_trend_ = .026). No associations were observed for ITC, social support, help during emergencies, comfort in faith and spirituality, and total mortality among Black men. The inverse association between depression scores and mortality is only seen among cases diagnosed within 5 years after study enrollment (Table S1) but not among those diagnosed after 5 years (Table S2).

**Table 3.** Association between psychosocial factors and total mortality by race.

	Model 1 adjustment					Model 2 adjustment		
	Black		White			Black	White	
Psychosocial variables	Events	aHR (95% CI)	Events	aHR (95% CI)	*P*_interaction (race)_	aHR (95%CI)	aHR (95%CI)	*P*_interaction (race)_
Depression								
No depressive symptoms	209	Referent	34	Referent	**.023**	Referent	Referent	**.021**
Probable depressive symptoms	60	0.77 (0.57 to 1.04)	11	1.59 (0.74 to 3.40)		0.74 (0.55 to 1.00)	1.33 (0.58 to 3.05)	
Major depressive symptoms	17	0.75 (0.46 to 1.24)	11	**2.62** (**1.17 to 5.86**)		0.67 (0.41 to 1.11)	**2.56** (**1.13 to 5.77**)	
*P*_trend_		0.073		**0.018**		**0.026**	**0.029**	
Continuous (SD increase)	286	0.89 (0.78 to 1.01)	56	**1.50** (**1.12 to 2.00**)		**0.84** (**0.74-0.96**)	**1.45** (**1.07 to 1.95**)	
Help during emergencies								
≥2 People	213	Referent	46	Referent	.597	Referent	Referent	.719
<2 People	74	0.99 (0.76 to 1.30)	12	0.62 (0.31 to 1.24)		0.94 (0.72 to 1.24)	0.66 (0.33 to 1.32)	
Support (friends/relatives)								
≥2 People	234	Referent	43	Referent	.344	Referent	Referent	.218
<2 People	54	0.82 (0.61 to 1.11)	14	1.03 (0.54 to 1.99)		0.78 (0.58 to 1.05)	1.23 (0.64 to 2.39)	
Inability to control important things in life								
Rarely or none of the time	174	Referent	23	Referent	**.042**	Referent	Referent	**.035**
Some of the time	84	0.80 (0.61 to 1.04)	19	1.84 (0.95 to 3.55)		**0.74** (**0.56-0.96**)	1.74 (0.89 to 3.40)	
much, most, or all of the time	32	0.97 (0.66 to 1.42)	14	**2.85** (**1.23 to 5.41**)		0.89 (0.61 to 1.32)	**2.30** (**1.07 to 4.95**)	
*P*_trend_		0.362		**0.010**		0.136	**0.028**	
Difficulties of things piling on and not overcoming them								
Rarely or none of the time	175	Referent	29	Referent	.221	Referent	Referent	.156
Some of the time	78	0.90 (0.68 to 1.18)	13	1.62 (0.79 to 3.33)		0.81 (0.61 to 1.07)	1.70 (0.82 to 3.51)	
Much, most, or all of the time	33	0.94 (0.64 to 1.39)	12	1.69 (0.77 to 3.70)		0.89 (0.60 to 1.30)	1.49 (0.65 to 3.40)	
*P*_trend_		0.566		0.143		0.253	0.242	
Comfort in faith								
A great deal	196	Referent	24	Referent	.964	Referent	Referent	.989
Quite a bit	67	0.99 (0.74 to 1.30)	15	1.02 (0.51 to 2.03)		1.03 (0.78 to 1.37)	1.06 (0.52 to 2.17)	
Not very much/somewhat	19	1.14 (0.76 to 1.71)	19	1.17 (0.63 to 2.17)		1.11 (0.74 to 1.67)	0.93 (0.49 to 1.78)	
*P*_trend_		0.663		0.640		0.621	0.853	
Attending faith-based services								
< Once per week	147	Referent	37	Referent	.428	Referent	Referent	.329
≥ Once per week	143	**0.77** (**0.61-0.98**)	22	1.00 (0.56 to 1.78)		0.84 (0.66 to 1.08)	1.41 (0.75 to 2.65)	
Spirituality								
Very	145	Referent	21	Referent	.712	Referent	Referent	.950
Fairly	99	1.18 (0.91 to 1.53)	24	0.92 (0.49 to 1.74)		1.15 (0.88 to 1.49)	1.02 (0.53 to 1.96)	
Slightly/not at all	45	1.28 (0.90 to 1.80)	14	1.31 (0.64 to 2.66)		1.32 (0.93 to 1.86)	1.02 (0.49 to 2.14)	
*P*_trend_		0.110		0.536		0.098	0.946	

Cox proportional hazards models were used for multivariable analyses. Model 1: age of PC diagnosis, time interval from enrollment to cancer diagnosis, education, income, cancer stage, prostate cancer treatment, enrollment source, comorbidity, and PSA history. Model 2: Model 1, plus physical activity, smoking, and daily alcohol consumption. SD (White = 5.68, Black = 4.94). All psychosocial variables were tested independently. Abbreviations: aHR = adjusted hazard ratio; CI = confidence interval. Bold indicates the association reached statistical significance.

Among White men self-reporting major depressive symptoms, the increased risk of total mortality was 2.6-fold (aHR = 2.56, 95% CI = 1.13 to 5.77, *P*_trend_ = .029) in addition to per standard deviation (5.68) increase in depressive symptoms (aHR = 1.45, 95% CI = 1.07 to 1.95) in fully adjusted models. In the fully adjusted model, the ITC continued to be associated with mortality (aHR = 2.30, 95% CI = 1.07 to 4.95, *P*_trend_ = .028 for “Much, Most, or all of the time”). No associations were observed between social support, help during emergencies, comfort in faith, attending faith-based services, spirituality, and total mortality among White men. The significant associations were mainly seen among patients diagnosed within 5 years after study enrollment for depression (aHR = 5.08, 95% CI = 1.41 to 18.32, *P*_trend_ = .19) and per SD increase of depression scores (aHR = 1.90, 95% CI = 1.20 to 3.00) (Tables S1 and S2).

Table 4 provides the impact of depression on total mortality after adjustment for psychosocial variables among White men. When psychosocial factors were additionally adjusted for individually and collectively, the associations with depression remained significant and some attenuated when controlling for those having help during emergencies (aHR = 3.11, 95% CI = 1.34 to 7.22), having close friends and relatives (aHR = 2.72, 95% CI = 1.12 to 6.64), having the difficulty of things piling on and not overcoming them (aHR = 2.93, 95% CI = 1.16 to 7.40), comfort in faith (aHR = 2.96, 95% CI = 1.27 to 6.91), attending faith-based services (aHR = 2.53, 95% CI = 1.12 to 5.69), and spirituality (aHR = 2.60, 95% CI = 1.14 to 5.94). Adjusting for ITC (aHR = 1.87, 95% CI = 0.72 to 4.84) attenuated the association. Adjusting for marital status (aHR = 2.27, 95% CI = 0.99 to 5.22), and adjustment of all psychosocial factors (aHR = 2.55, 95% CI = 0.94 to 6.96) resulted in an association that was no longer significant, but did not substantially change the point estimate. When assessing these relationships with depression as continuous in multivariable models ITC was the only psychosocial factor that attenuated the relationship between depressive symptoms and the risk of total mortality (aHR = 1.30, 95% CI = 0.90 to 1.88 for per SD increase in depressive symptom score), while all other estimates remained statistically significant. Among patients diagnosed <5 years since study enrollment, estimates adjusted for all psychosocial factors except ITC remained significant. The depression-death association adjusted for ITC lost its significance (aHR = 1.70, 95% CI = 0.95 to 3.05 for per SD increase in depressive symptom score) (Table S1). No associations were observed for patients diagnosed ≥5 years after study enrollment (Table S3).

**Table 4.** Association between depression and total mortality among White men with additional adjustment for other psychosocial variables.

	Depression among White men							
Model (M)	None, *n* = 34 (<10)	Probable, *n* = 11 (10-15)		Major depression, *n *= 11 (≥16)		*P*_trend_	Continuous (SD increase)	
Full adj.	Referent	1.33	0.58-3.05	**2.56**	**1.13-5.77**	**.029**	**1.45**	**1.07-1.95**
Additional adjustment								
M1: Help during emergencies	Referent	1.47	0.64-3.38	**3.11**	**1.34-7.22**	**.010**	**1.54**	**1.14-2.08**
M2: Close friends/relatives	Referent	1.34	0.58-3.07	**2.72**	**1.12-6.64**	**.034**	**1.46**	**1.07-2.00**
M3: Inability to control important things in life	Referent	1.02	0.41-2.53	1.87	0.72-4.84	.237	1.30	0.90-1.88
M4: Difficulties of things piling on and not overcoming them	Referent	1.37	0.55-3.41	**2.93**	**1.16-7.40**	**.028**	**1.59**	**1.12-2.27**
M5: Comfort in faith	Referent	1.40	0.62-3.18	**2.96**	**1.27-6.91**	**.015**	**1.51**	**1.11-2.05**
M6: Attending faith-based services	Referent	1.31	0.57-3.03	**2.53**	**1.12-5.69**	**.031**	**1.44**	**1.07-1.94**
M7: Spirituality	Referent	1.32	0.57-3.05	**2.60**	**1.14-5.94**	**.029**	**1.45**	**1.07-1.95**
M8: Marital status	Referent	1.16	0.50-2.70	2.27	0.99-5.22	.071	**1.40**	**1.03-1.89**
M9: All additional adjustment	Referent	1.15	0.44-2.97	2.55	0.94-6.96	.088	**1.54**	**1.05-2.24**

Cox proportional hazard models were used for multivariable analyses. Full model (age of PC diagnosis, time interval from enrollment to cancer diagnosis, education, income, cancer stage, prostate cancer treatment, enrollment source, comorbidity, PSA history, physical activity, smoking, and daily alcohol consumption) and further adjustment for: M1 (help during emergencies), M2 (close friends/relatives), M3 (inability to control important things in life), M4 (difficulties of things piling on and not overcoming them), M5 (comfort in faith), M6 (attending faith-based services), M7 (spirituality), M8 (marital status), and M9 (M1-M8); SD (White = 5.68). Bold indicates the association reached statistical significance.

Table 5 shows the depression category—and depression score—total mortality associations after further adjustment for psychosocial factors among Black men. A significant inverse association was observed for per SD increase of depression score, even when other psychosocial factors were adjusted for, individually or collectively. The inverse depression score-mortality association was restricted to patients diagnosed within 5 years after study enrollment (Table S3).

**Table 5.** Association between depression and total mortality among Black men with additional adjustment for other psychosocial variables.

	Depression among Black men							
Model (M)	None, *n* = 209 (<10)	Probable, *n* = 60 (10-15)		Major depression, *n* = 17 (≥16)		*P*_trend_	Continuous (SD increase)	
Full adj.	Referent	**0.74**	**0.55-1.00**	0.67	0.41-1.11	**.026**	**0.84**	**0.74-0.96**
Additional adjustment								
M1: Help during emergencies	Referent	0.74	0.55-1.01	0.68	0.41-1.12	**.028**	**0.84**	**0.74-0.96**
M2: Close friends/relatives	Referent	0.75	0.56-1.02	0.71	0.43-1.18	**.044**	**0.85**	**0.75-0.97**
M3: Inability to control important things in life	Referent	0.77	0.55-1.08	0.65	0.37-1.14	.063	**0.84**	**0.72-0.98**
M4: Difficulties of things piling on and not overcoming them	Referent	0.75	0.54-1.04	0.63	0.36-1.12	**.042**	**0.82**	**0.70-0.96**
M5: Comfort in faith	Referent	**0.73**	**0.54-0.99**	0.65	0.39-1.08	**.018**	**0.83**	**0.73-0.95**
M6: Attending faith-based services	Referent	**0.74**	**0.55-1.00**	0.67	0.40-1.10	**.024**	**0.84**	**0.74-0.95**
M7: Spirituality	Referent	**0.73**	**0.54-0.99**	0.65	0.39-1.07	**.017**	**0.83**	**0.72-0.94**
M8: Marital status	Referent	**0.73**	**0.54-0.99**	0.66	0.40-1.09	**.019**	**0.83**	**0.73-0.94**
M9: All additional adjustment	Referent	0.76	0.54-1.07	0.64	0.35-1.16	.067	**0.82**	**0.69-0.96**

Cox proportional hazards models were used for multivariable analyses. Full model (age of PC diagnosis, time interval from enrollment to cancer diagnosis, education, income, cancer stage, prostate cancer treatment, enrollment source, comorbidity, PSA history, physical activity, smoking, and daily alcohol consumption) and further adjustment for: M1 (help during emergencies), M2 (close friends/relatives), M3 (inability to control important things in life), M4 (difficulties of things piling on and not overcoming them), M5 (comfort in faith), M6 (attending faith-based services), M7 spirituality), M8 (marital status), and M9 (M1-M8); SD (Black = 4.94). Bold indicates the association reached statistical significance.

The underlying/contributing cause of PCa deaths was 13 among White men and 98 among Black men. No statistically significant associations were observed between depression, help during emergencies, close friends and relatives, ITC, difficulties controlling for important things in life, comfort in one’s faith, attending faith-based services, and spirituality among both White and Black men in fully adjusted competing risk models.

## Discussion

Psychosocial factors and their effect on total mortality among men with PCa were evaluated in this cohort of predominantly low-income Black and White participants. We found that Black men who attended faith-based services had a reduced risk of total mortality compared with those not attending faith-based services; however, the association no longer remained after controlling for physical activity, smoking, and daily alcohol consumption. Depression score was inversely associated with total mortality among Black PCa patients. In contrast, White PCa patients with depressive symptoms or the ITC were at higher risk of total mortality than those without. The associations with depression were primarily seen among patients who were diagnosed with 5 years since study enrollment and attenuated when further adjusting for the ITC.

Growing evidence on depression and mortality among Black men with PCa^4-9^ has shown that even among high-stress occupations such as military service,^20^ both White and Black men with PCa were at increased risk of depression and all-cause mortality, though it was slightly higher among Black men.^20^ However, in our study of predominantly low-income men, we found that depressive symptoms were significantly associated with reduced mortality among Black men with PCa but increased mortality among White patients, which does not correspond with prior epidemiologic findings.^4-9^ To eliminate the possibility of a false finding, we carefully adjusted a wide arrange of confounders and examined whether the association could be accounted for by other psychosocial factors by adjusting for them individually and collectively. The depression-mortality associations remained largely unchanged. We further explored associations by analyzing the data stratified by time interval between study enrollment and PCa diagnosis (<5 years and ≥5 years). We found the associations were mainly restricted among patients who were diagnosed close to study enrollment when the misclassification error of depression measurement is lower. It should be noted that vast majority of deaths in our study cohort, particularly for White subjects, were attributed to causes other than PCa. When PCa specific mortality was considered, no significant association was observed among both Black and White subjects although these analyses were seriously under powered.

Although speculative, there are some plausible explanations for our findings. Black men with depressive symptoms may face greater mental health stigma than those without depressive symptoms, which may influence responses to sensitive questions. A scoping review by Bamidele et al.^21^ suggested that “a complex intersection of self-stigma, public stigma, and structural stigma affected Black men’s perceptions on their masculinity,” which can be influenced by their illness status. For example, structural stigma may lead to avoidance of the topic of PCa and treatment side-effects in social discussions, as well as marital insecurity. Likewise, self-stigma may induce embarrassment, and as mentioned, masculinity concerns regarding screening procedures (eg, rectal examination).^21^ Additionally, public stigma may present as victim-blaming (eg, “perceived diagnosis resulting from reckless living”) or being stereotyped by others.^21^ Major implications for stigma, in general, include avoidance of screening, participating in research, and social communication.^21^ Because this population is predominately a low-income cohort, stigma may be even greater compared with those with higher income. Unfortunately, stigma toward mental health was not assessed in the parent study. Future research should evaluate stigma and its impact toward masculinity concerns and mental health among men with PCa particularly among low-income populations.

In addition, this study observed an inverse association with mortality for attending faith-based services at least once per week among Black men. These findings correspond with prior epidemiologic studies that also evaluated spiritual and religious beliefs and practices on PCa survival.^12^^,^^15^ However, these associations were no longer significant when controlling for physical activity, smoking, and daily alcohol consumption, where physical activity had the greatest impact. Physical activity is known not only to increase one’s mobility (ie, attending faith-based services) but also to improve PCa survival.^22^^,^^23^ Although their spiritual beliefs and practices were reported at baseline, it is possible that one’s religious beliefs and practices may change or be challenged as a result of a PCa diagnosis. This could have resulted in misclassification of religious beliefs during the period of diagnosis and survival. For example, a study evaluating the relationship between race and religious coping in the diagnosis and decision of PCa treatment found that Black men had either improved or deviated from their religious faith.^24^ Compared with White men, Bowie et al. observed that Black men were more likely to consider that their cancer was a punishment from God (82% more likely), that cancer tested their faith (99% more likely), and that their faith weakened after diagnosis (36% more likely). However, compared with White men, Black men also reported believing that their faith increased since diagnosis (81% more likely), that their cancer was cured with enough prayer (3.6-fold more likely), and that a cure is God’s decision (2.2-fold more likely).^24^ Similar epidemiologic patterns were observed for depressive symptoms among men with PCa, yet we found no statistically significant associations by racial groups.^25^ When further adjusting for additional psychosocial and potential protective factors in White men, the depressive symptom and total mortality association was attenuated when controlling for the ITC (highest impact), supporting its important role in depression and mortality.^14^

Our study has several strengths, including ascertainment of psychosocial factors, demographics, lifestyle factors, and PCa survivorship in a large, predominately low-income population comprising two-thirds Black men. However, there are limitations to consider. First, psychosocial factors were ascertained at baseline; therefore, it is possible that participants had changes in depressive symptoms, spiritual beliefs and practices, support structure from friends and family, and their ability to maintain normalcy when confronted with a PCa diagnosis. These may have biased point estimates toward the null, particularly among patients diagnosed long after study enrollment (time of exposure assessment). Additionally, as mental health is not easily discussed, it is possible that social desirability and information biases may be present and differential by race, which may affect responses to some questions in Black men. Although this study had adjusted for many potential confounders, unmeasured confounding may remain. For example, not ascertaining stigma toward mental health may affect the way participants respond to survey questions or seek help; thus, our ability to control for it in multivariable models or assess potential multiplicative interactions was compromised. Additionally, we were not able to control for accessing mental health facilities due to the limited responses in its use. Although our study included the largest number of low-income PCa patients thus far, the statistical power is still not optimal, particularly for variables other than depression and ITC. For example, our study only has 80% statistical power to detect the minimal detectable HRs of 1.73 to 1.89 for comfort in faith, and 0.71 and 0.55 for help during emergencies and Black, and White subjects. Furthermore, due to a small number of events, PCa-specific mortality analysis was seriously underpowered, particularly for analyses stratifying by race. Thus, the PCa-specific mortality was not considered as the primary outcome of the study. Last, multiple comparisons were performed in our study. Because many of the psychosocial variables are correlated, we opted not to adjust for number of comparisons. Thus, chance finding cannot be ruled out. Future studies with larger sample sizes are needed to evaluate depression and psychosocial factors on PCa specific mortality and should consider both competing risks effect modification by race.

Behavioral health research and the impact on incidence and survivorship (for all cancers) must be prioritized. Results from our study support calls for more research on psychological disorders among men with PCa to improve treatment efficacy, PCa risk, and quality of life.^8^^,^^26^ Additionally, Cuevas et al.^26^ also emphasize the limited studies, challenges, and future opportunities evaluating PCa racial disparities within a psychosocial context, including socioeconomic status, segregation, psychological factors, emotional regulation, psychological well-being/resilience, and how racial groups are affected.

## Conclusion

We observed elevated total mortality among White men with PCa self-reporting depressive symptoms and an ITC while depression score was inversely associated with total mortality among Black patients. Results from this study support the need to evaluate potential psychosocial dynamics that may improve survival among men with PCa and how personal coping strategies, whether positive or negative, influence survivorship. In addition, future research should assess the impact of psychotherapy and patient treatment from clinicians toward survivorship. Such evaluation should consider individuals’ demographic backgrounds. Additionally, future research should account for support systems like family, friends, or spouse, and spiritual and religious beliefs and practices, as well as coping with mental health, stress, and stigma to reduce barriers to seeking treatment.

## Supplementary Material

pkag029_Supplementary_Data

## Data Availability

Data used in this study can be accessed via an application to the Southern Community Cohort Study’s Data and Biospecimen Use Committee (www.southerncommunitystudy.org).
