# Supplementary material for: Psychosocial factors and overall mortality after prostate cancer diagnosis among predominantly low-income Black and White adults
Source: JNCI Cancer Spectr. 2026 May 5;10(3):pkag029. doi: 10.1093/jncics/pkag029 (PMC13166870; doi:10.1093/jncics/pkag029)
Supplement: pkag029_Supplementary_Data [file pkag029_supplementary_data.docx]

**Supplementary Material**

| **Table S1: Association between psychosocial factors and total mortality by race among patients diagnosed <5 years after study enrollment.** | | | | | | | | |
| --- | --- | --- | --- | --- | --- | --- | --- | --- |
|  | **Model 1 Adjustment** | | | | | **Model 2 Adjustment** | | |
|  |  | **Black** |  | **White** |  | **Black** | **White** |  |
| **Psychosocial Variables** | **Events** | **aHR**  **95%CI** | **Events** | **aHR**  **95%CI** | **p_interaction_**  **_(race)_** | **aHR**  **95%CI** | **aHR**  **95%CI** | **p_interaction_**  **_(race)_** |
| **Depression** |  |  |  |  |  |  |  |  |
| *No depressive symptoms* | 109 | Ref. | 18 | Ref. | **0.032** | Ref. | Ref. | **0.031** |
| *Probable depressive symptoms* | 26 | 0.55  0.35-0.87 | 7 | 1.87  0.69-5.06 |  | 0.55  0.35-0.88 | 1.65  0.58-4.72 |  |
| *Major depressive symptoms* | 10 | 0.91  0.46-1.80 | **7** | **4.46**  **1.31-15.16** |  | 0.79  0.40-1.56 | **5.08**  **1.41-18.32** |  |
| *p-trend* |  | 0.094 |  | **0.017** |  | 0.056 | **0.019** |  |
| *Continuous (SD Increase)* | 145 | **0.80**  **0.66-0.97** | 32 | **1.89**  **1.23-2.91** |  | **0.75**  **0.62-0.91** | **1.90**  **1.20-3.00** |  |
| **Help during emergencies** |  |  |  |  |  |  |  |  |
| *≥2 People* | 113 | Ref. | 22 | Ref. | 0.297 | Ref. | Ref. | 0.277 |
| *<2 People* | 33 | 0.99  0.66-1.47 | 12 | 1.07  0.41-2.78 |  | 0.96  0.64-1.44 | 1.22  0.47-3.18 |  |
| **Support (Friends/Relatives)** |  |  |  |  |  |  |  |  |
| *≥2 People* | 118 | Ref. | 22 | Ref. | 0.143 | Ref. | Ref. | 0.085 |
| *<2 People* | 27 | 0.90  0.59-1.38 | 12 | 1.62  0.69-3.79 |  | 0.83  0.54-1.28 | 1.95  0.81-4.68 |  |
| **Inability to Control Important Things in Life (ITC)** |  |  |  |  |  |  |  |  |
| *Rarely or none of the time* | 96 | Ref. | 11 | Ref. | **0.011** | Ref. | Ref. | **0.006** |
| *Some of the time* | 38 | **0.63**  **0.42-0.95** | 12 | **2.44**  **1.00-5.95** |  | **0.56**  **0.37-0.84** | 2.41  0.96-6.07 |  |
| *Much, Most, or all of the time* | 12 | 0.87  0.47-1.63 | 10 | **3.32**  **1.20-9.16** |  | 0.75  0.40-1.40 | 2.70  0.93-7.84 |  |
| *p-trend* |  | 0.128 |  | **0.015** |  | **0.034** | **0.048** |  |
| **Difficulties of things Piling on and not Overcoming them** |  |  |  |  |  |  |  |  |
| *Rarely or none of the time* | 92 | Ref. | 14 | Ref. | **0.049** | Ref. | Ref. | **0.028** |
| *Some of the time* | 36 | 0.81  0.54-1.21 | 8 | 2.17  0.83-5.68 |  | 0.72  0.48-1.08 | 2.11  0.77-5.74 |  |
| *Much, Most, or all of the time* | 17 | 1.59  0.92-2.72 | 10 | **3.40**  **1.20-9.63** |  | 1.34  0.77-2.30 | 2.87  0.97-8.48 |  |
| *p-trend* |  | 0.482 |  | **0.015** |  | 0.982 | **0.043** |  |
| **Comfort in Faith** |  |  |  |  |  |  |  |  |
| *A great deal* | 99 | Ref. | 13 | Ref. | 0.233 | Ref. | Ref. | 0.464 |
| *Quite a bit* | 37 | 1.21  0.82-1.79 | 8 | 0.80  0.30-2.16 |  | 1.28  0.86-1.91 | 0.78  0.27-2.25 |  |
| *Not very much / Somewhat* | 11 | 0.81  0.43-1.54 | 13 | 1.63  0.69-3.85 |  | 0.81  0.42-1.55 | 1.14  0.46-2.81 |  |
| *p-trend* |  | 0.984 |  | 0.303 |  | 0.933 | 0.812 |  |
| **Attending Faith-Based Services** |  |  |  |  |  |  |  |  |
| *< Once per week* | 75 | Ref. | 22 | Ref. | 0.241 | Ref. | Ref. | 0.126 |
| *≥ Once per week* | 71 | **0.70**  **0.49-0.99** | 13 | 1.18  0.51-2.74 |  | 0.77  0.54-1.10 | 1.84  0.71-4.76 |  |
| **Spirituality** |  |  |  |  |  |  |  |  |
| *Very* | 77 | Ref. | 13 | Ref. | 0.719 | Ref. | Ref. | 0.976 |
| *Fairly* | 50 | 1.10  0.76-1.59 | 15 | 0.89  0.38-2.09 |  | 1.05  0.72-1.52 | 0.96  0.41-2.25 |  |
| *Slightly / Not at all* | 18 | 0.98  0.58-1.68 | 7 | 1.36  0.48-3.82 |  | 1.09  0.64-1.87 | 0.91  0.30-2.83 |  |
| *p-trend* |  | 0.870 |  | 0.662 |  | 0.715 | 0.872 |  |
| *Cox Proportional Hazard Models were used for multivariable analyses. Model 1: Age of PC Diagnosis, time interval of enrollment to cancer diagnosis, education, income, cancer stage, prostate cancer treatment, enrollment source, comorbidity, PSA ever; Model 2: Model 1, physical activity, smoking, and daily alcohol consumption. SD (White = 5.68, Black = 4.94). All psychosocial variables were tested independently.* | | | | | | | | |

| **Table S2: Association between psychosocial factors and total mortality by race among patients diagnosed ≥5 years after study enrollment** | | | | | | | | |
| --- | --- | --- | --- | --- | --- | --- | --- | --- |
|  | **Model 1 Adjustment** | | | | | **Model 2 Adjustment** | | |
|  | **Black** | | **White** | |  | **Black** | **White** |  |
| **Psychosocial Variables** | **Events** | **aHR**  **95%CI** | **Events** | **aHR**  **95%CI** | **p_interaction_**  **_(race)_** | **aHR**  **95%CI** | **aHR**  **95%CI** | **p_interaction_**  **_(race)_** |
| **Depression** |  |  |  |  |  |  |  |  |
| *No depressive symptoms* | 100 | Ref. | 16 | Ref. | 0.290 | Ref. | Ref. | 0.253 |
| *Probable depressive symptoms* | 34 | 0.99  0.66-1.48 | 4 | 1.37  0.38-4.95 |  | 0.94  0.62-1.41 | 0.74  0.16-3.54 |  |
| *Major depressive symptoms* | 7 | 0.60  0.27-1.31 | 4 | 1.98  0.53-7.34 |  | 0.54  0.24-1.20 | 1.66  0.44-6.33 |  |
| *p-trend* |  | 0.320 |  | 0.303 |  | 0.186 | 0.548 |  |
| *Continuous (SD Increase)* | 141 | 0.96  0.80-1.15 | 24 | 1.19  0.74-1.91 |  | 0.92  0.77-1.11 | 1.10  0.67-1.81 |  |
| **Help during emergencies** |  |  |  |  |  |  |  |  |
| *≥2 People* | 100 | Ref. | 24 | Ref. | - | Ref. | Ref. | - |
| *<2 People* | 41 | 0.97  0.66-1.42 | 0 | - |  | 0.94  0.64-1.39 | - |  |
| **Support (Friends/Relatives)** |  |  |  |  |  |  |  |  |
| *≥2 People* | 116 | Ref. | 21 | Ref. | 0.455 | Ref. | Ref. | 0.491 |
| *<2 People* | 27 | 0.71  0.46-1.10 | 2 | 0.35  0.08-1.60 |  | 0.70  0.45-1.07 | 0.41  0.09-1.92 |  |
| **Inability to Control Important Things in Life (ITC)** |  |  |  |  |  |  |  |  |
| *Rarely or none of the time* | 78 | Ref. | 12 | Ref. | 0.686 | Ref. | Ref. | 0.671 |
| *Some of the time* | 46 | 0.88  0.60-1.28 | 7 | 1.32  0.47-3.70 |  | 0.82  0.56-1.19 | 1.15  0.38-3.52 |  |
| *Much, Most, or all of the time* | 20 | 0.99  0.59-1.64 | 4 | 2.02  0.55-7.48 |  | 0.93  0.56-1.55 | 1.86  0.47-7.38 |  |
| *p-trend* |  | 0.752 |  | 0.298 |  | 0.526 | 0.415 |  |
| **Difficulties of things Piling on and not Overcoming them** |  |  |  |  |  |  |  |  |
| *Rarely or none of the time* | 83 | Ref. | 15 | Ref. | 0.970 | Ref. | Ref. | 0.976 |
| *Some of the time* | 42 | 0.92  0.63-1.34 | 5 | 0.96  0.28-3.23 |  | 0.85  0.58-1.26 | 0.95  0.28-3.22 |  |
| *Much, Most, or all of the time* | 16 | 0.63  0.36-1.10 | 2 | 0.48  0.09-2.57 |  | 0.60  0.34-1.05 | 0.29  0.04-2.19 |  |
| *p-trend* |  | 0.124 |  | 0.435 |  | 0.072 | 0.316 |  |
| **Comfort in Faith** |  |  |  |  |  |  |  |  |
| *A great deal* | 97 | Ref. | 11 | Ref. | 0.320 | Ref. | Ref. | 0.278 |
| *Quite a bit* | 30 | 0.89  0.58-1.34 | 7 | 1.04  0.37-2.94 |  | 0.91  0.60-1.39 | 1.12  0.39-3.19 |  |
| *Not very much / Somewhat* | 17 | 1.50  0.88-2.56 | 6 | 0.64  0.23-1.83 |  | 1.49  0.87-2.56 | 0.62  0.19-1.98 |  |
| *p-trend* |  | 0.388 |  | 0.446 |  | 0.370 | 0.475 |  |
| **Attending Faith-Based Services** |  |  |  |  |  |  |  |  |
| *< Once per week* | 72 | Ref. | 15 | Ref. | 0.964 | Ref. | Ref. | 0.994 |
| *≥ Once per week* | 72 | 0.89  0.63-1.25 | 9 | 1.06  0.42-2.66 |  | 0.94  0.67-1.33 | 1.28  0.48-3.42 |  |
| **Spirituality** |  |  |  |  |  |  |  |  |
| *Very* | 68 | Ref. | 8 | Ref. | 0.513 | Ref. | Ref. | 0.647 |
| *Fairly* | 49 | 1.28  0.88-1.87 | 9 | 1.07  0.36-3.16 |  | 1.22  0.83-1.80 | 1.37  0.43-4.36 |  |
| *Slightly / Not at all* | 27 | 1.40  0.88-2.22 | 7 | 1.51  0.49-4.67 |  | 1.39  0.88-2.22 | 1.45  0.46-4.64 |  |
| *p-trend* |  | 0.107 |  | 0.507 |  | 0.132 | 0.502 |  |
| *Cox Proportional Hazard Models were used for multivariable analyses. Model 1: Age of PC Diagnosis, time interval of enrollment to cancer diagnosis, education, income, cancer stage, prostate cancer treatment, enrollment source, comorbidity, PSA ever; Model 2: Model 1, physical activity, smoking, and daily alcohol consumption. SD (White = 5.68, Black = 4.94). All psychosocial variables were tested independently.* | | | | | | | | |

| **Table S3: Association between depression score and total mortality with additional adjustment for other psychosocial variables, stratified time window between study enrollment and prostate cancer diagnosis** | | | | |
| --- | --- | --- | --- | --- |
|  | **Among White Men** | | **Among Black Men** | |
| **Model (M)** | *Depression score*  *(SD Increase)*  *<5years* | *Depression score*    *(SD Increase)*  *≥5years* | *Depression score*    *(SD Increase)*  *<5years* | *Depression score*  *(SD Increase)*  *≥5years* |
| **Full Adj.** | **1.90**  **1.20-3.00** | 1.10  0.67-1.81 | **0.75**  **0.62-0.91** | 0.92  0.77-1.11 |
| **Additional Adjustment** |  |  |  |  |
| M1: Help during emergencies | **1.99**  **1.22-3.24** | 1.28  0.80-2.06 | **0.75**  **0.62-0.91** | 0.92  0.77-1.11 |
| M2: Close friends/relatives | **1.91**  **1.15-3.17** | 1.17  0.71-1.93 | **0.76**  **0.62-0.92** | 0.94  0.78-1.13 |
| M3: Inability to control important things in life (ITC) | 1.70  0.95-3.05 | 1.05  0.59-1.88 | **0.80**  **0.65-0.99** | 0.91  0.73-1.14 |
| M4: Difficulties of things Piling on and not  Overcoming them | **2.04**  **1.09-3.83** | 1.37  0.80-2.35 | **0.68**  **0.54-0.86** | 1.02  0.81-1.28 |
| M5: Comfort in faith | **1.95**  **1.21-3.14** | 1.14  0.69-1.88 | **0.75**  **0.61-0.91** | 0.90  0.74-1.08 |
| M6: Attending faith-based services | **1.85**  **1.16-2.94** | 1.12  0.68-1.86 | **0.75**  **0.62-0.91** | 0.91  0.76-1.10 |
| M7: Spirituality | **1.97**  **1.22-3.18** | 1.10  0.67-1.83 | **0.75**  **0.62-0.91** | 0.90  0.74-1.08 |
| M8: Marital status | **1.83**  **1.13-2.96** | 1.07  0.64-1.78 | **0.74**  **0.61-0.89** | 0.91  0.76-1.10 |
| M9: All Additional Adjustment | **2.73**  **1.15-6.48** | 1.60  0.84-3.05 | **0.69**  **0.54-0.87** | 0.97  0.76-1.23 |
| *Cox Proportional Hazard Models were used for multivariable analyses. Full model (Age of PC Diagnosis, time interval of enrollment to cancer diagnosis, education, income, cancer stage, prostate cancer treatment, enrollment source, comorbidity, PSA ever, physical activity, smoking, and daily alcohol consumption) and further adjustment for: M1 (Help during emergencies); M2 (Close friends/relatives); M3 (Inability to control important things in life “ITC”); M4 (Difficulties of things Piling on and not Overcoming them); M5 (Comfort in faith); M6 (Attending faith-based services); M7 Spirituality); M8 (Marital status); M9 (M1-M8); SD (White = 5.68).* | | | | |
